# Isolation of Mutants of the Nitrogen-Fixing Actinomycete *Frankia*

**DOI:** 10.1264/jsme2.ME13126

**Published:** 2013-12-28

**Authors:** Kentaro Kakoi, Masatoshi Yamaura, Toshihito Kamiharai, Daiki Tamari, Mikiko Abe, Toshiki Uchiumi, Ken-Ichi Kucho

**Affiliations:** 1 Graduate School of Science and Engineering, Kagoshima University, Korimoto 1–21–35, Kagoshima 890–0065, Japan; 2 Department of Chemistry and Bioscience, Faculty of Science, Kagoshima University, Korimoto 1–21–35, Kagoshima 890–0065, Japan

**Keywords:** 5-fluoroorotic acid, next generation sequencer, nitrogen fixation, symbiosis, uracil auxotroph

## Abstract

*Frankia* is a nitrogen (N)-fixing multicellular actinomycete which establishes root-nodule symbiosis with actinorhizal plants. Several aspects of *Frankia* N fixation and symbiosis are distinct, but genes involved in the specific features are largely unknown because of the lack of an efficient mutant screening method. In this study, we isolated mutants of *Frankia* sp. strain CcI3 using hyphae fragments mutagenized by chemical mutagens. Firstly, we isolated uracil auxotrophs as gain-of-function mutants resistant to 5-fluoroorotic acid (5-FOA). We obtained seven 5-FOA resistant mutants, all of which required uracil for growth. Five strains carried a frame shift mutation in orotidine-5′-phosphate decarboxylase gene and two carried an amino acid substitution in the orotate phosphoribosyltransferase gene. Secondly, we isolated mutants showing loss-of-function phenotypes. Mutagenized hyphae were fragmented by ultrasound and allowed to multiply at their tips. Hyphae were fragmented again and short fragments were enriched by filtration through 5 μm pores filters. Next-generation and Sanger sequencing revealed that colonies formed from the short hyphae fragments consisted of cells with an identical genotype. From the mutagenized colony population, we isolated three pigmentation mutants and a mutant with reduced N-fixation activity. These results indicate that our procedure is useful for the isolation of loss-of-function mutants using hyphae of *Frankia*.

Nitrogen is an essential element for plant growth. Although nitrogen gas accounts for 78% of the atmosphere, most organisms cannot assimilate the dinitrogen molecule because of its stable triple bond. Nitrogen-fixing (N-fixing) bacteria have the ability to reduce dinitrogen to ammonium, which is available to plants as well as bacteria and fungi. Some N-fixing bacteria establish symbioses with plants, thus enhancing their growth on N-deficient soil. Two groups of symbiotic N-fixing bacteria, rhizobia and *Frankia*, colonize root nodules, a specialized organ for symbiosis formed on the host plant’s roots.

Rhizobia are Gram-negative bacteria that establish symbiosis with leguminous plants. The molecular underpinnings of the interaction between plant and bacteria have been well studied in the symbiosis. Rhizobia produce lipo-chitooligosaccharides called Nod factor, which function as a signaling molecule that allows its host plant to recognize a compatible symbiont (9). The *nod* genes are involved in synthesis and secretion of the Nod factor (9) and are clustered in a particular region of the genome, the symbiosis island, (13) or in a symbiosis plasmid (10). Specific flavonoids released from the host plant’s root regulate Nod factor production with mediation of the NodD transcription factor (9). Moreover, surface polysaccharides of rhizobia are also important in the establishment of successful symbiosis (3, 21).

*Frankia* is a genus of Gram-positive multicellular actinomycetes, which are phylogenetically separate from rhizobia. It is able to fix N_2_ (even in free-living conditions) by developing a vesicle, which is a spherical cell devoted to N fixation. The vesicle is surrounded by several tens of hopanoid lipid envelopes that function as a barrier for oxygen, which inactivates nitrogenase (4). *Frankia* establishes symbiosis with non-legume plant species (>200) belonging to 23 genera of 8 families called actinorhizal plants (16). Genome analysis of *Frankia* strains identified sets of genes known to be related to N fixation but neither a convincing *nod* gene cluster nor a significant symbiosis island was found (1, 16, 17). These results suggest that *Frankia* does not use rhizobia-like *nod* genes and Nod factors to communicate with actinorhizal plants. Homologues of genes in the hopanoid biosynthesis pathway were also found in the *Frankia* genome (16) but those involved in the differentiation and development of vesicle cells have not been identified. Genomes reveal a wealth of valuable biological information but identification of novel genes, which are usually related to unique features of specific organisms, is limited by homology-based annotation.

Forward genetics is a powerful approach generally used to identify genes involved in a certain biological function but it is not yet feasible in *Frankia*. Forward genetic analysis starts with screening mutants that are defective in the biological function of interest. Then, the mutated gene responsible for the defect is identified by functional complementation with a wild-type gene. However, it is difficult to isolate mutants of multicellular bacteria such as *Frankia* because a single colony formed from hyphae contains multiple cells with different genotypes. Even if a colony partially contains mutant cells, the mutant phenotype is not apparently observed because such a recessive phenotype is masked by a dominant phenotype expressed by the remaining wild-type cells in the colony. Use of spores or protoplasts is a general solution for this problem. Generation of protoplasts and spores has been reported for several *Frankia* strains (7, 22), but model *Frankia* strains whose complete genome sequence has been determined (16) poorly regenerate protoplasts and poorly germinate spores, thus rendering them impractical for forward genetics. Therefore, in this study, we attempted to isolate *Frankia* mutants using multicellular hyphae.

## Materials and Methods

### Bacterial strain and growth condition

*Frankia* sp. strain CcI3 (24) was grown at 28°C in the media described below. Kanamycin (25 μg mL^−1^) and cycloheximide (10 μg mL^−1^) were added to avoid contamination with bacteria and mold.

### Isolation of uracil auxotrophs with 5-fluoroorotic acid (5-FOA)

*Frankia* CcI3 cells grown in BAP-T medium (14) were collected from 80 mL culture by centrifugation at 25,000×*g* and resuspended in 7 mL BAP-T. Hyphae were homogenized by forced passage through a 21G needle (TERUMO, Tokyo, Japan) six times. We added ethyl methanesulfonate (EMS) to 1 mL cell suspensions to final concentrations of 1, 2, 4 or 8% and incubated them for 9 min. Cells were collected by centrifugation at 21,500×*g* for 1 min and resuspended in 1 ml 5% (w/v) sodium thiosulfate to inactivate EMS. Cells were collected by centrifugation and washed twice with sterile distilled water. We incubated the cells in 1 mL BAP-T medium containing 100 μg mL^−1^ uracil and 100 μg mL^−1^ uridine for 2 days. The cell suspensions were plated on solid CB media (2) containing 0.5 mg mL^−1^ 5-FOA, 100 μg mL^−1^ uracil and 100 μg mL^−1^ uridine, and incubated for 2 months. To determine CFU, 50 μl aliquots of 10^−2^, 10^−3^ and 10^−4^ dilutions of cell suspension were plated on solid CB media.

### Phenotype analysis of uracil auxotrophs

A 5-FOA resistant (5-FOA^R^) colony was placed in a 1.5-mL tube with 100 μl sterile distilled water and homogenized with a pestle (Scientific Specialties, Lodi, CA, USA). The homogenate was plated on CB medium containing 0.5 mg mL^−1^ 5-FOA and incubated until colonies formed. A single colony was picked up and grown on the same 5-FOA medium again. Then, a single colony was inoculated into 3 mL liquid CB medium containing 100 μg mL^−1^ uracil and cultured with shaking at 200 rpm. Hyphae were homogenized by forced passage through a 21G needle and grown in 10 ml BAP-T medium containing 100 μg mL^−1^ uracil in a 25-cm^2^ tissue culture flask (TPP, Trasadingen, Switzerland). Hyphae were homogenized and diluted to a concentration of OD_660_ = 0.05. Ten microliters of the homogenate were spotted on solid CB medium lacking Bacto Proteose Peptone No. 3 (BD Biosciences, Sparks, MD, USA) (CB minimal medium), CB minimal medium containing 100 μg mL^−1^ uracil, and CB minimal medium containing 100 μg mL^−1^ uracil and 0.5 mg mL^−1^ 5-FOA, and the plates were incubated.

### Generation of colonies consisting of cells with an identical phenotype

*Frankia* CcI3 hyphae grown in BAP-T medium (2 mL) were transferred to a 15-mL centrifuge tube (Greiner, Tokyo, Japan) and fragmented using a SoniMix ultrasonic homogenizer UX-050 (Mitsui Electric, Chiba, Japan) with an output power setting of 50% for 10 s. Tip (3-mm diameter) was located at half-depth of the hyphae suspension. The fragmented hyphae from 1 mL culture were mutagenized by proflavin or EMS. Cells were resuspended in 12 or 24 mg mL^−1^ proflavin, incubated for 1 h and washed five times with sterile distilled water. Alternatively, cells were resuspended in 2% EMS and incubated for 9 min. Cells were collected by centrifugation for 1 min and resuspended in 5% (w/v) sodium thiosulfate. Cells were collected by centrifugation and washed twice with sterile distilled water. The mutagenized CcI3 cells were inoculated into 10 mL liquid CB media and grown until cell density increased 5 to 10 times. Two milliliters of the culture were transferred to a 15-mL centrifuge tube (Greiner) and homogenized by ultrasound as described above. Five hundred microliters of the homogenized hyphae were transferred to Ultrafree centrifugal filter units (5 μm pore; Millipore, Billerica, MA, USA) and centrifuged at 12,000×*g* for 1 min. The filtrate was spread onto solid CB media and incubated to generate colonies.

### Detection of mutations by next-generation sequencing

We resequenced genomes of mutant *Frankia* strains using the next-generation sequencer SOLiD 4 system (Applied Biosystems, Foster City, CA, USA). *Frankia* cells were collected from 10 mL culture and genomic DNA was purified using the DNeasy Plant Mini Kit (Qiagen, Hilden, Germany) or by the procedure described by Kucho *et al.* (14). Libraries were generated using the 5500 SOLiD fragment library core kit (Applied Biosystems) with 1 μg genomic DNA. Template beads were prepared using the SOLiD ePCR kit V2 (Applied Biosystems) and the SOLiD XD beads enrichment kit (Applied Biosystems). Beads were deposited on a glass slide using the SOLiD XD slide & deposition kit v2 (Applied Biosystems). Fifty base pairs at the ends of library fragments were sequenced using the SOLiD ToP fragment BC sequencing kit (Applied Biosystems) and the SOLiD ToP instrument buffer kit (Applied Biosystems). All the experiments were performed according to the manufacturers’ instructions.

The 50-bp reads were mapped on a reference genome sequence of *Frankia* CcI3 (accession No. NC_007777) and single nucleotide polymorphisms (SNPs) were detected using Bioscope software (Applied Biosystems). We accepted only SNP calls with a “het flag” of zero, which indicates that a statistically significant portion of reads showed a homogenous mutation at that nucleotide position. Several SNPs were detected even in the wild-type strain CcI3 maintained in our laboratory, but they are not included in Table S2.

### Confirmation of mutations by Sanger method

Templates for sequencing were amplified by PCR from genomic DNA using primer sets listed in Table S1 and *Ex Taq* (Takara Bio, Otsu, Japan) with GC buffer I (Takara Bio) or PrimeSTAR DNA polymerase (Takara Bio). PCR products were electrophoresed and purified from the gel using a Genclean kit (Q-Biogene, Carlsbad, CA, USA) or reacted with exonuclease I (Takara Bio) and shrimp alkaline phosphatase (Roche, Mannheim, Germany) to remove residual primers and dNTPs. Sequencing reactions were performed with the purified DNA, one of the primers in Table S1 and the BigDye terminator v3.1 cycle sequencing kit (Applied Biosystems). Products of the sequencing reactions were purified using Agencourt CleanSEQ (Beckman Coulter, Brea, CA, USA) and chromatograms were obtained by ABI PRISM 3130xl Genetic Analyzer (Applied Biosystems).

### Screening for nitrogen-fixation mutants

Eight-strip PCR tubes (Watson, Kobe, Japan) were filled with 75 μl of 1-mm diameter glass beads (As One, Osaka, Japan), two particles of 2-mm diameter glass beads (As One) and 100 μl sterile distilled water. A *Frankia* colony was picked up and placed in each tube, and tubes were vigorously agitated by Vortex-Genie2 (Scientific Industries, Bohemia, NY, USA) for 5 min to homogenize the colony clump. Two microliters of the homogenates were spotted onto solid CB minimal medium lacking NH_4_Cl (CBminN-). Five microliters of the homogenates were inoculated into 96-well microtiter plates containing 100 μl liquid CB minimal media. Strains that did not grow on CBminN- medium but grew in CB minimal medium were recovered and inoculated again into solid CBminN- and liquid CB media. Strains that grew on CBminN-medium at comparable levels to wild-type CcI3 were discarded. The remaining strains were recovered, washed with sterile distilled water and inoculated onto solid CB and CBminN- media. Strains that grew on CBminN- medium at comparable levels to wild-type CcI3 or that showed apparently reduced growth on CB medium were discarded.

### Measurement of acetylene reduction activity (ARA)

*Frankia* cells cultured to the mid-log phase in liquid CB medium were collected by centrifugation and washed twice with CBminN-medium. Hyphae were homogenized by a 21G needle and inoculated at a concentration of OD_660_ = 0.03 to 90 mL liquid CB minimal and CBminN-media in a culture bottle (VIDREX, Fukuoka, Japan). After 6 to 9 days, 5 mL of the culture was transferred to a 7-mL vacutainer (BD Biosciences) and 10% (v/v) acetylene was injected. After 4-h incubation at 28°C, 1 mL gas phase was analyzed by gas chromatograph (GC8-AIF; Shimadzu, Kyoto, Japan) to quantify the amount of ethylene produced. We extracted total protein from the *Frankia* cells by heating them at 90°C in 1 N NaOH for 15 min as described previously (22) and determined the concentration with Protein Assay (Bio-Rad, Hercules, CA, USA) using bovine serum albumin as a standard. Ethylene generated in 1 h by cells equivalent to 1 mg total protein was represented as ARA.

## Results

### Isolation of gain-of-function mutants

We first screened for uracil auxotrophs as gain-of-function mutants that show resistance to 5-FOA (5, 11, 20). We expected that mutants with such a dominant phenotype would be easily isolated even when multicellular hyphae were used for screening. The 5-FOA, a fluorinated analog of orotic acid, is converted to 5-fluorouridine monophosphate (5-FUMP) by two reactions catalyzed by orotate phosphoribosyltransferase (PyrE) and orotidine-5′-phosphate decarboxylase (PyrF) (Fig. S1). Since 5-FUMP is toxic, the wild-type strain cannot grow in the presence of 5-FOA. Mutants of the *pyrE* or *pyrF* gene show resistance to 5-FOA but they cannot convert orotic acid to uridine monophosphate (UMP), which is essential for nucleic acid synthesis. Growth of the mutants, however, can be supported by supplementing the medium with uracil; thus, we can isolate uracil auxotrophs as 5-FOA^R^ mutants.

**Fig. 1:**
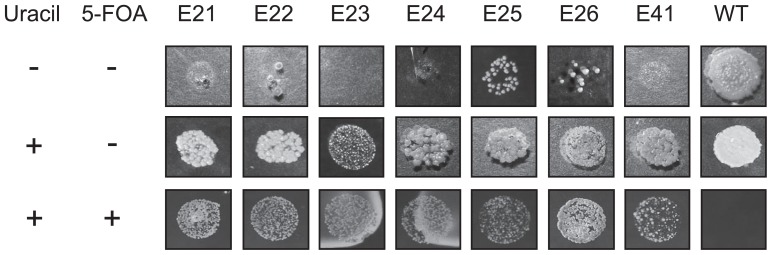
Uracil auxotrophy and 5-FOA resistance of the mutants. Mutants and the wild type (WT) were grown on CB minimal medium alone (top), supplemented with uracil (middle) or supplemented with uracil and 5-FOA (bottom).

Hyphae of *Frankia* CcI3 were mutagenized by various concentrations of EMS (1, 2, 4 and 8%). The survival rate of the cells decreased as the concentration of EMS increased (Table 1), indicating that EMS was effective. Seven 5-FOA^R^ colonies were observed (Table 1 and Fig. 1); six (E21, E22, E23, E24, E25, and E26) were from hyphae mutagenized by 2% EMS and the other (E41) was from those mutagenized by 4% EMS.

Strains E21, E23, E24 and E41 exhibited clear uracil auxotrophy; they did not grow on minimal medium but showed comparable growth to the wild-type strain when supplemented with uracil (Fig. 1). In contrast, several colonies appeared when strains E22, E25 and E26 were grown on minimal medium, with frequencies of 2.0×10^−4^, 8.3×10^−4^ and 4.0×10^−4^, respectively.

We amplified the *pyrE* and *pyrF* genes of the mutants by PCR and determined their nucleotide sequences. As expected, all mutants carried a mutation in one of the two genes (Table 2). Strains E22 and E24 carried a mutation in *pyrE* and strains E21, E23, E25, E26 and E41 carried a mutation in *pyrF*. The *pyrE* mutants had a nucleotide substitution that caused an amino acid change. A 1-bp deletion in a stretch of guanines was found in *pyrF* mutants E21, E23 and E41. Insertion of a guanine at the same G-stretch was found in the other *pyrF* mutants, E25 and E26. Both the deletion and the insertion resulted in a frame shift.

### Isolation of loss-of-function mutants

**Fig. 2:**
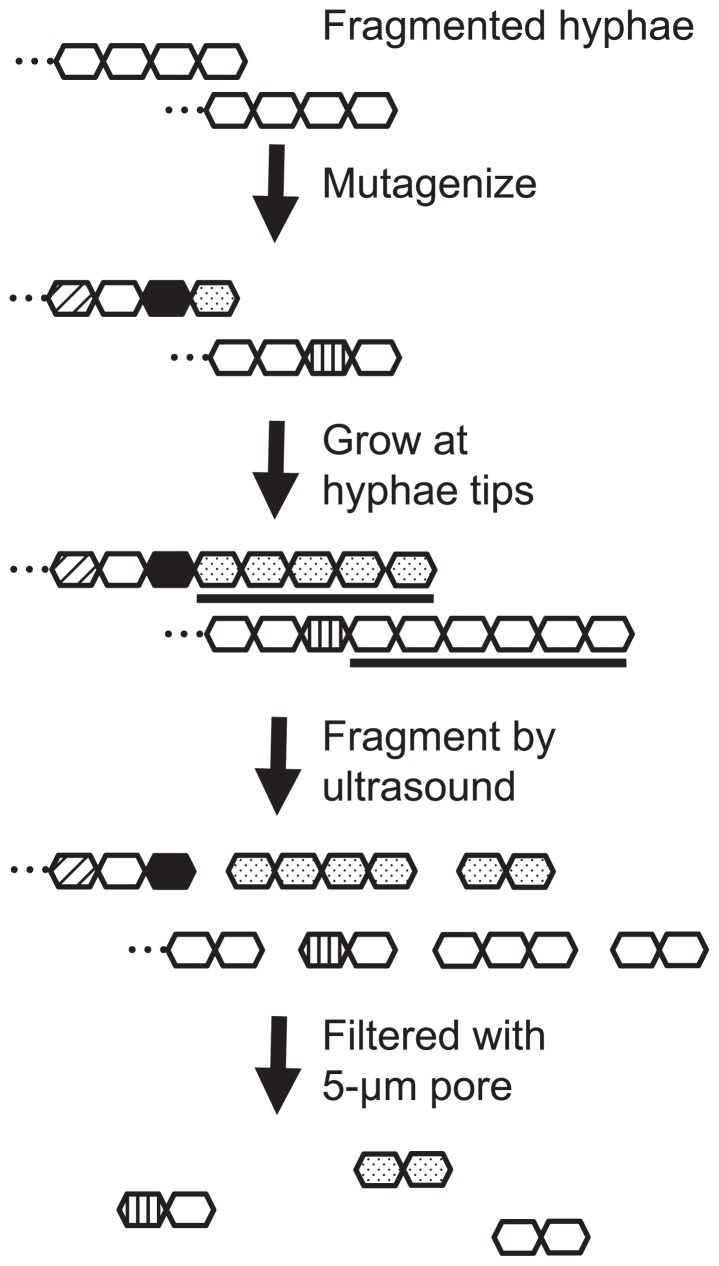
Procedure to enrich hyphae fragments consisting of cells with an identical genotype. White hexagons indicate wild-type cells; hexagons patterns indicate different mutant genotypes; bars indicate cells that are derived from a tip cell and carry an identical genotype.

Since the loss-of-function phenotype is recessive, it is not apparently expressed if a colony is formed from multiple cells with heterogeneous genotypes. Therefore, to isolate loss-of-function mutants, it is essential to generate homogenous hyphae fragments which consist of only cells with an identical genotype. We attempted to achieve this goal by making use of the property of *Frankia* that hyphae grow at their tips (Fig. 2), although sometimes they ramify. Fragmented hyphae were mutagenized and the tip cells were allowed to divide several times in liquid medium. At this step, cells with an identical genotype are arranged at the tips of hyphae. These hyphae were fragmented again. Short hypha fragments released from the tips contained only cells with an identical genotype and these were enriched by filtration with 5-μm pores.

The dominant length of the filtrated hypha fragments was 8 to 12 μm (Fig. S2A). Since the length of a *Frankia* CcI3 cell is about 2.4 μm (Fig. S2B), the majority of the filtrated hypha fragments were estimated to consist of 3 to 5 cells.

**Fig. 3:**
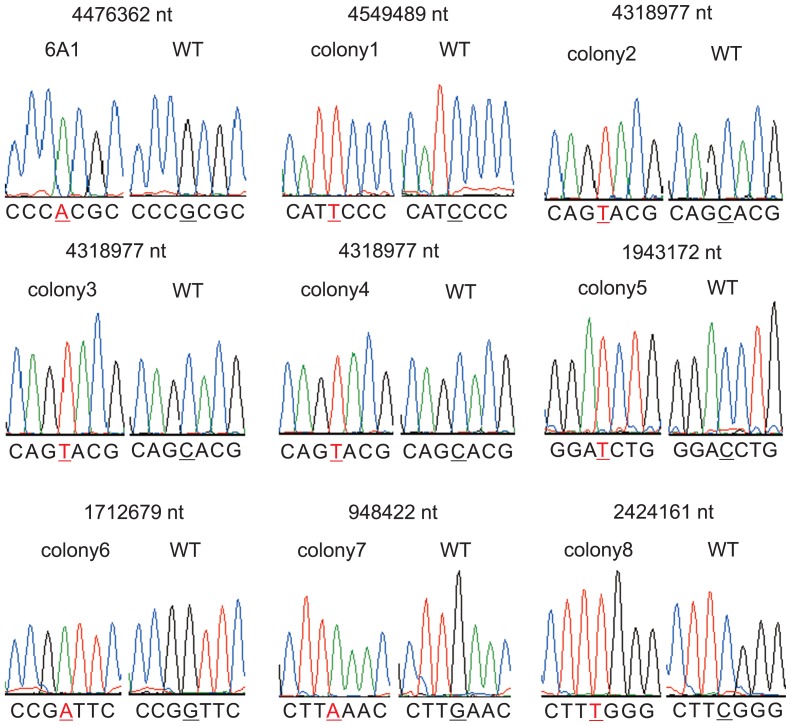
Chromatograms obtained by Sanger sequencing. Nine representative results of 38 analyzed mutations are shown. The mutated base and its corresponding base in the wild type (WT) are underlined. The position of the base in the genome sequence is shown above the chromatogram. Chromatograms were obtained using Sequence Scanner ver.1.0 (Applied Biosynthesis).

To confirm if the method worked as expected, filtered hyphae were grown to colonies on CB complete medium and their genome sequences were analyzed by a next-generation sequencer. We analyzed genomes of 13 colonies; nine (colony 1 to colony 9) showed no visible phenotype and four (6A1 and *shiro1* to *shiro3*, described below) showed mutant phenotypes. For each colony, sequence data equivalent to about 200-times coverage of the genome were obtained. In all of the colonies, substitution mutations, at which most reads indicated a mutated base, were detected (Table S2). The number of mutations was 9 to 20 per genome. A small portion of reads indicated a wild-type base (Table S2) but they were likely to be caused by random sequencing errors. For validation, we amplified genomic regions including a mutation by PCR and directly determined the nucleotide sequence of the products by the Sanger method (19). Thirty-eight mutations in 10 colonies were analyzed. The Sanger and next-generation sequencing methods detected identical mutations (Fig. 3). Importantly, multiple peaks were not detected at the locations of the mutated bases, suggesting that each colony consists of only a single mutant cell line.

**Fig. 4:**
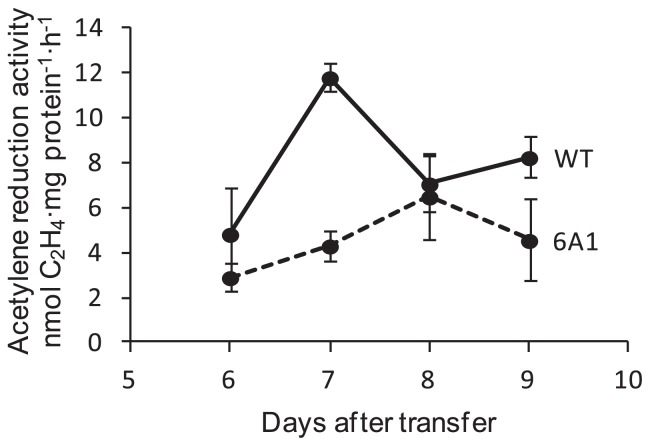
Acetylene reduction activity of 6A1 and the wild type (WT) during 6 to 9 days after transfer to CBminN- media. Dot and bar represent the average and SE, respectively, of three independent determinations.

As a trial to isolate mutants with a loss-of-function phenotype, we screened for N-fixation mutants. We replicated 2,400 colonies in liquid CB minimal and solid CBminN-media. As a result of multiple rounds of screening, we isolated a mutant strain 6A1, which grew slightly slower than the wild type on solid CBminN- media. Strain 6A1 showed substantial N fixation activity (ARA) in CBminN- medium but at significantly lower levels than the wild type (Fig. 4). The result was reproduced in three series of experiments (Fig. S3).

**Fig. 5:**
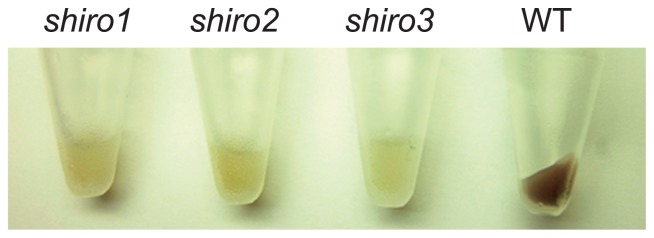
Photographs of pigmentation mutants and the wild type (WT). Each mutant was grown in CB liquid media and harvested in a microtube by centrifugation.

*Frankia* CcI3 produces a brown pigment when it is cultivated in CB medium. Among the 2,400 colonies we found three white colonies that did not accumulate the pigment (*shiro1*, *shiro2* and *shiro3*, “shiro” means white in Japanese) (Fig. 5). The *shiro1* and *shiro2* originated from EMS-treated cells and the *shiro3* was from proflavin-treated cells. The phenotype was stably inherited during subculture. Taken together, these results demonstrate the usefulness of our method for the isolation of loss-of-function mutants.

## Discussion

### Gain-of-function mutants

Two previous articles reported the isolation of gain-of-function mutants using mutagens; Myers and Tisa (15) isolated numerous antibiotic-resistant and antimetabolite-resistant mutants using EMS and UV, and Carú and Cabello (8) isolated antibiotic-resistant mutants using *N*-methyl-*N*′-nitro-*N*-nitrosoguanidine. As in the former case, we also successfully isolated mutants using EMS; thus, this compound is an effective mutagen for *Frankia*. Several of the mutants isolated by Myers and Tisa lacked uracil phosphoribosyltransferase activity (an enzyme of the pyrimidine metabolic pathway), but they did not identify mutations in genes encoding the enzyme (15). In the present study, we identified nucleotide substitutions, deletions, and insertions in the genes responsible for uracil auxotrophy using genome information (Table 2). These are the first *Frankia* mutants whose phenotype has been characterized at the nucleotide sequence level.

When *pyrE* mutant E22 was grown on uracil-deficient minimal medium, a few colonies appeared (Fig. 1). Because the mutation in the *pyrE* gene was maintained in those colonies (data not shown), strain E22 would be a leaky mutant. The mutated amino acid is located outside of important functional domains such as substrate binding and active sites (data not shown), which would explain the weak phenotype.

The five *pyrF* mutants were classified into two allelic variants (Table 2). Strains E21, E23 and E41 carried a deletion that causes a frame shift downstream of the 132th amino acid and translation of the mutant PyrF terminates at the 133th amino acid (data not shown). This results in deletion of five of seven substrate binding sites, which explains the obvious uracil auxotrophy of these mutants. Strains E25 and E26 carried insertion of a guanine in the same stretch of guanines as the deletion mutants (Table 2). Such stretches of identical nucleotides would be subject to insertion and deletion in the *Frankia* genome.

Stable transformation has not been achieved in *Frankia* yet (14). These uracil auxotroph mutants are useful as a host strain for transformation using wild-type *pyr* genes as a marker. Such native genes show promise for efficient expression in highly GC-rich *Frankia*.

### Loss-of-function mutants

Although nine colonies were picked up independently of any visible phenotypes without selective pressure, all carried mutations in their genomes (Table S2). The high occurrence of mutant colonies indicates that most cells accumulated mutations under the mutagenesis conditions we used. In principle, our procedure does not completely exclude colonies with a heterogeneous genotype (Fig. 2) but none of the nine colonies apparently showed such a genotype (Table S2 and Fig. 3). The ratio of the initial length of hyphae used for mutagenesis to the length of the newly elongated hyphae at their tips affects heterogeneity (Fig. 2); the larger the ratio, the more heterogeneous colonies appear. Our experimental conditions would give a sufficiently small ratio to suppress the occurrence of heterogeneous colonies to a workable level. Most colonies obtained by our procedure appeared to consist of a homogenous population of mutant cells, thus enabling the screening of loss-of-function mutants. In addition, the fact that genotypically homogenous colonies were obtained without selective pressure suggests that *Frankia* hyphae are not multinuclear, like those of *Streptomyces* (12).

Proflavin is known to cause either nucleotide insertions or deletions (6) but we detected only one deletion in one strain mutagenized by proflavin (data not shown). Therefore, proflavin may be an ineffective mutagen for *Frankia*. It is also possible that the next-generation sequencer SOLiD system failed to detect indels because of its lower sensitivity to indels than other platforms (18).

We isolated four mutants from the population of 2,400 mutagenized colonies. Three (*shiro1*, *shiro2* and *shiro3*) were pigmentation mutants that showed an obvious change in cell color (Fig. 5). The brown pigment could be melanin because a *Frankia* strain is reported to produce the compound (23). As *shiro1* and *shiro2* shared two identical mutations in *Francci3_2727* and *Francci3_2745* (Table S2), these mutations are potentially responsible for the aberrant phenotype. *Francci3_2745* shows homology to a putative lysylphosphatidylglycerol biosynthesis bifunctional protein, although it was originally annotated as hypothetical in the genome database. It may therefore have relevance to melanin biosynthesis. The other, strain 6A1, showed defects in N fixation in ammonium-free minimal medium but the activity was not completely abolished (Figs. 4 and S3), suggesting that the mutations were not critical and that the resulting proteins retained substantial function. No mutations in the strain 6A1 genome were found in known N-fixation-related genes (Table S2). We will be able to isolate mutants with more obvious phenotypes by screening a larger population. At present, we are not able to identify the mutations responsible for the loss-of-function mutant phenotypes because stable transformation of *Frankia* is not feasible. Establishment of the transformation method will enable to identify such mutations by genetic complementation of the mutants.

In most cases, mutants defective in biologically important features, such as N fixation and symbiosis with plants, are of a loss-of-function type. Our procedure can be applied to screening a wide range of mutant types and will greatly contribute to the molecular genetics of *Frankia*.

## Supplementary material



## Figures and Tables

**Table 1:** Survival rate of CcI3 cells exposed to EMS

EMS conc. (%)	Titer (× 10^6^ CFU)	Survival rate (%)	5-FOA^R^ colony
0	3.2	100	0
1	2.4	73	0
2	2.2	69	6
4	1.5	47	1
8	0.7	22	0

**Table 2:** Mutations found in *pyrE* and *pyrF* genes

Strain	Gene	Sequence^a^	Amino acid change
Wild type	*pyrE*	254 CCCGGTT 260	
E22	*pyrE*	254 CCCaGTT 260	R86Q
Wild type	*pyrE*	113 TCGAAGC 119	
E24	*pyrE*	113 TCGgAGC 119	E39G
Wild type	*pyrF*	385 GCTGGGG 391	
E21, E23, E41	*pyrF*	385 GCT-GGG 390	Frame shift
E25, E26	*pyrF*	385 GCTgGGGG 392	Frame shift

a A lower case letter indicates nucleotide substitution or insertion; a hyphen indicates deletion; numbers indicate position of the nucleotide relative to the first position of the translation initiation codon.
