# Supplementary material for: Isolation of Mutants of the Nitrogen-Fixing Actinomycete Frankia
Source: Microbes Environ. 2013 Dec 28;29(1):31–7. doi: 10.1264/jsme2.ME13126 (PMC4041240; doi:10.1264/jsme2.ME13126)
Supplement: Supplementary file 1 [file 29_31_s1.pdf]

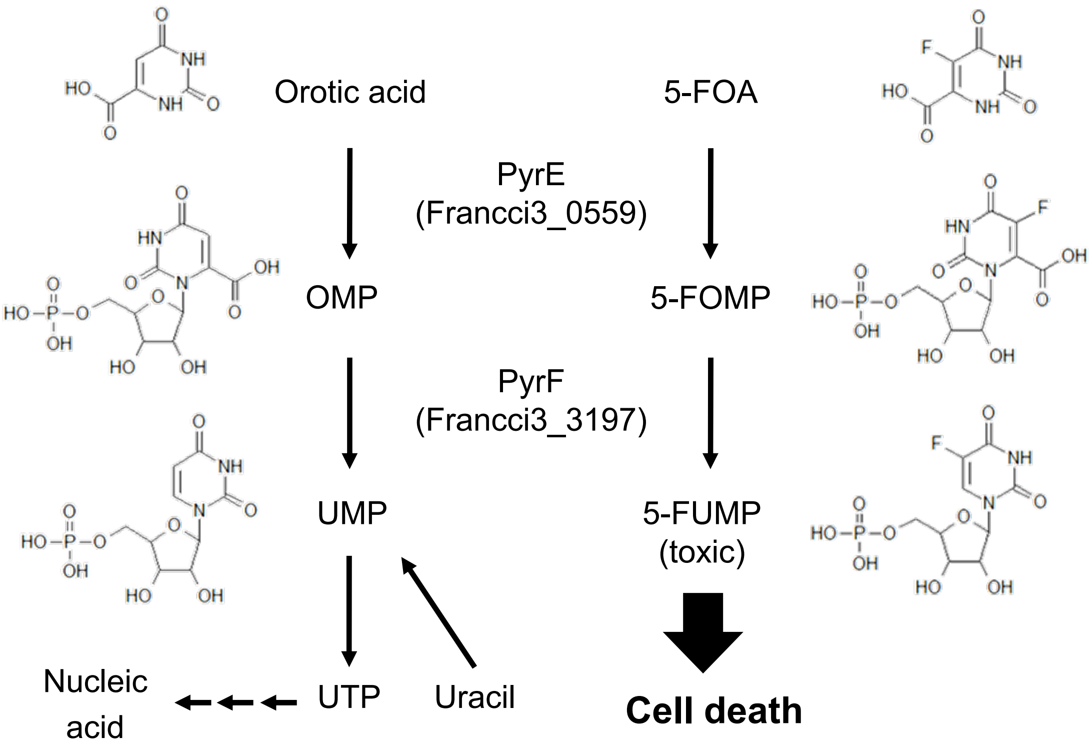

Fig. S1. Schematic diagram of metabolism of orotic acid and its fluorinated analog (5-FOA).

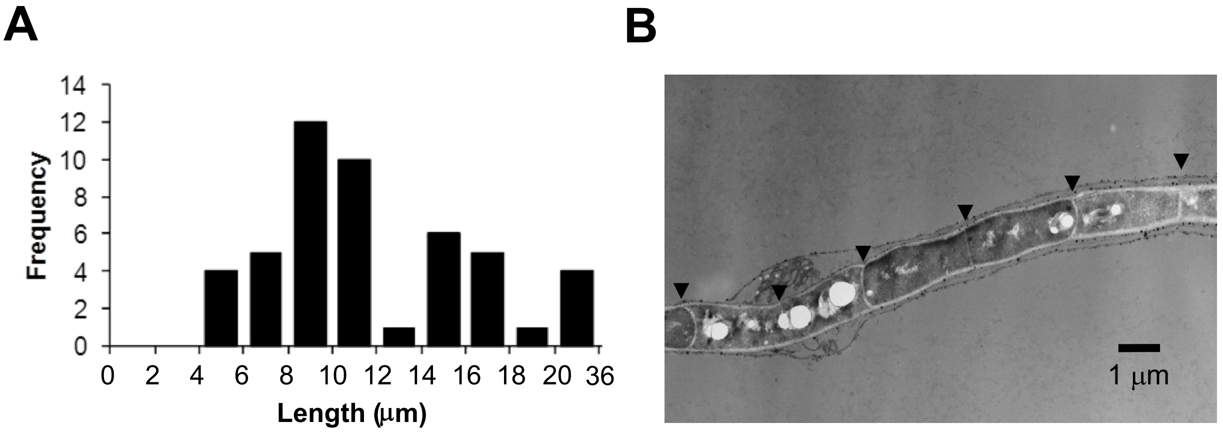

Fig. S2. (A) Histogram of length of filtrated hyphae (n = 48). (B) Hyphae of *Frankia* CcI3 observed by transmission electron microscopy (TEM). Septum is indicated by arrowhead.

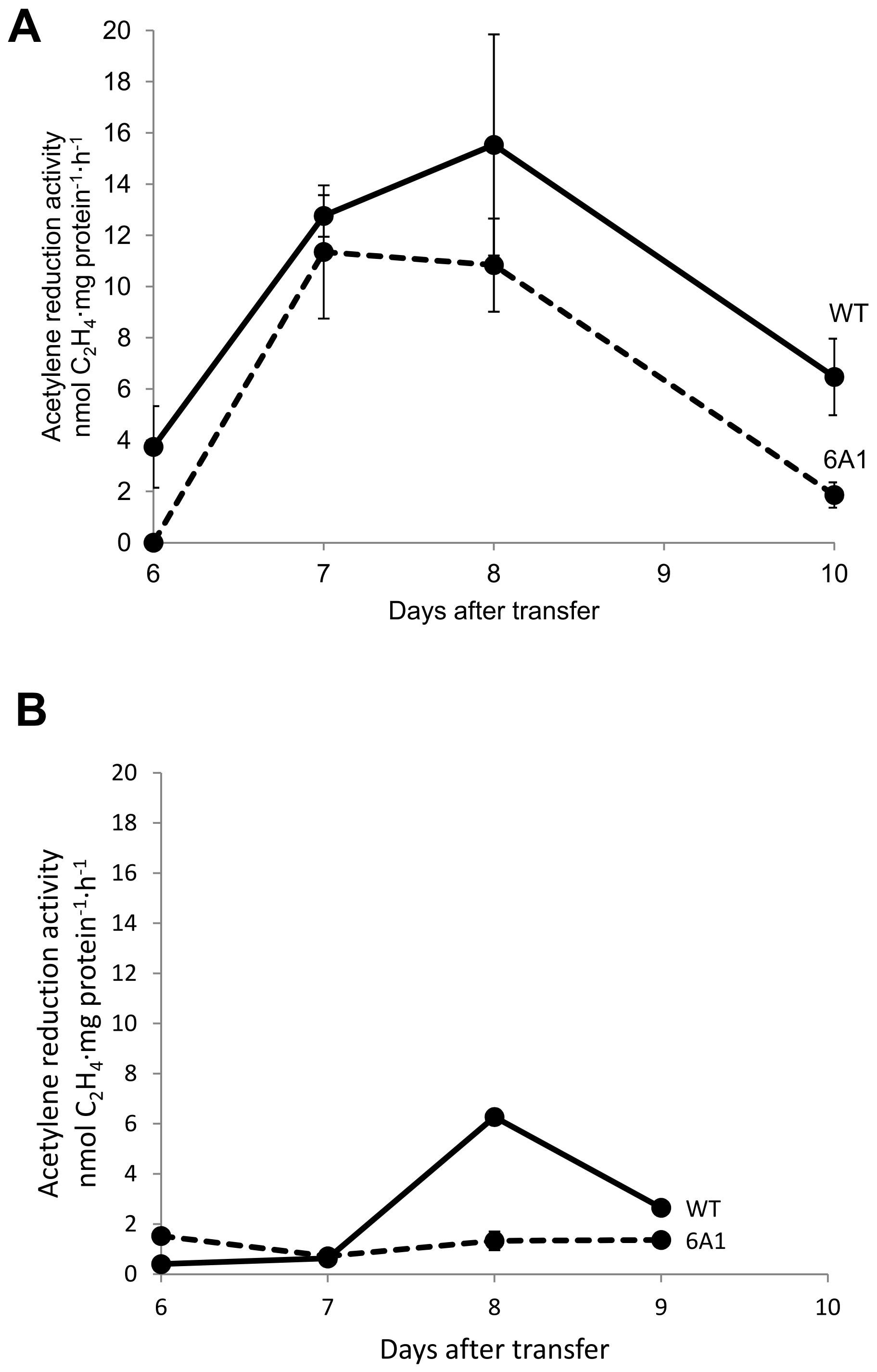

Fig. S3. Acetylene reduction activity of 6A1 and wild type (WT) during 6 to 10 days after transfer to CBminN- media. The dot and bar represent average and SE, respectively, from three independent determinations. Results from two additional experiments are shown (A and B).

Table S1. List of primers used in this study.

Strain	Direction	Position[a]	Length	Sequence(5' to 3')
pyrE mutant	Forward	650818	20	ATCATGCTTCTACACGGGCT
pyrE mutant	Reverse	651528	19	GATGACATGAGGCAACGGT
pyrF mutant	Forward	3787819	20	AAGGCTTTGACGGACGAAAG
pyrF mutant	Reverse	3788902	20	ATCGTTGCAGGAATACCACG
6A1	Forward	4476075	15	CTTCGCGATAGCCGA
6A1	Reverse	4476548	15	CCAGCGAGTCGTTGA
colony1	Forward	4549220	17	CCGAAGGTGCCAAGGTA
colony1	Reverse	4549687	17	TTCCCTGGATCAAGCTG
colony2,3,4	Forward	4318791	18	GACGATCAACAGGGCACC
colony2,3,4	Reverse	4319424	18	ACCTGGACGGTTGGACAG
colony5	Forward	1942918	16	GCGTTGCTCCAGCCCC
colony5	Reverse	1943406	15	CTGGCGGCGTCGTAC
colony6	Forward	1712456	16	GGAGATCTTGAGGACG
colony6	Reverse	1712951	16	CAGACCTGCGGCGAAG
colony7	Forward	948210	15	ACCACTCGGCGGCAG
colony7	Reverse	948749	18	GCGCAGTTGATGATCTCG
colony8	Forward	2423961	15	CGCTGAGCGTCGCCA
colony8	Reverse	2424501	17	CGGCAGACTGGTGGGCA

[a]Position of 5' end of CcI3 genome sequence (NC_007777) to which a primer anneals.

Table S2. Mutations detected by next generation sequencing.

Strain	Position (nt)[a]	Mutant base	WT base[b]	Coverage[c]	No. WT base[d]	Ratio of WT base[e]	ID[f]	Annotation
6A1	200831	T	C	1225	52	0.042	IGR|Francci3_0169-Francci3_0170	
6A1	1011977	T	A	11	0	0.000	IGR|Francci3_0868-Francci3_0869	
6A1	1172483	A	G	179	4	0.022	Francci3_0989	carbamoyl-phosphate synthase L chain, ATP-binding
6A1	1212645	A	G	397	16	0.040	Francci3_1017	hypothetical protein
6A1	1986198	T	C	891	46	0.052	Francci3_1652	NADPH-dependent FMN reductase
6A1	3340080	T	C	41	1	0.024	Francci3_2835	protein tyrosine/serine phosphatase
6A1	4262098	A	G	816	23	0.028	IGR|Francci3_3562-Francci3_3563	
6A1	4476362	A	G	91	0	0.000	Francci3_3740	diguanylate cyclase/phosphodiesterase
6A1	4508907	A	G	420	24	0.057	Francci3_3760	signal transduction histidine kinase
colony1	3444812	T	C	159	4	0.025	IGR|Francci3_2923-Francci3_2924	
colony1	3610715	T	C	287	5	0.017	Francci3_3042	acyl-CoA dehydrogenase-like
colony1	3809698	C	T	4	0	0.000	Francci3_3216	hypothetical protein
colony1	3916026	T	C	20	2	0.100	Francci3_3305	Type IV secretory pathway VirD4 components-like
colony1	4384188	T	C	267	6	0.022	IGR|Francci3_3662-Francci3_3663	
colony1	4549489	A	G	117	8	0.068	Francci3_3796	peptidase M16-like
colony1	4630236	T	C	40	1	0.025	Francci3_3866	NUDIX hydrolase
colony1	4649421	T	C	310	10	0.032	IGR|Francci3_3885-Francci3_3886	
colony1	4814994	T	C	683	9	0.013	Francci3_4027	LuxR family transcriptional regulator
colony2	113563	A	C	9	1	0.111	Francci3_0092	hypothetical protein
colony2	214881	A	G	230	0	0.000	Francci3_0182	glyoxalase/bleomycin resistance protein/dioxygenase
colony2	245012	A	G	608	8	0.013	IGR|Francci3_0205-Francci3_0207	
colony2	755715	T	C	96	2	0.021	Francci3_0660	geranylgeranyl reductase
colony2	909997	T	C	31	0	0.000	Francci3_0781	type II secretion system protein
colony2	1116519	T	C	401	5	0.012	Francci3_0947	FHA domain-containing protein
colony2	1251205	C	G	6	0	0.000	Francci3_1052	hypothetical protein
colony2	1776439	A	G	69	1	0.014	Francci3_1484	cysteine dioxygenase type I
colony2	1991103	C	T	6	1	0.167	Francci3_1657	glycerol kinase
colony2	2377470	G	T	4	0	0.000	Francci3_2022	transcriptional regulator
colony2	2440216	A	G	67	1	0.015	Francci3_2075	aminotransferase
colony2	2801764	T	C	151	2	0.013	Francci3_2413	major facilitator transporter
colony2	2910081	A	G	651	11	0.017	IGR|Francci3_2466-Francci3_2467	

Strain	Position (nt)[a]	Mutant base	WT base[b]	Coverage[c]	No. WT base[d]	Ratio of WT base[e]	ID[f]	Annotation
colony2	4075193	T	C	244	8	0.033	Francci3_3427	hypothetical protein
colony2	4217197	T	C	483	6	0.012	IGR|Francci3_3528-Francci3_3530	
colony2	4318977	T	C	286	2	0.007	Francci3_3617	diguanylate cyclase/phosphodiesterase
colony2	4902347	A	G	254	8	0.031	Francci3_4093	NUDIX hydrolase
colony2	4921610	T	C	374	4	0.011	Francci3_4118	hypothetical protein
colony2	4983255	C	T	4	1	0.250	Francci3_4179	hypothetical protein
colony2	5290936	G	C	4	1	0.250	Francci3_4425	serine/threonine protein kinase
colony3	755715	T	C	161	6	0.037	Francci3_0660	geranylgeranyl reductase
colony3	1116519	T	C	769	10	0.013	Francci3_0947	FHA domain-containing protein
colony3	1776439	A	G	128	2	0.016	Francci3_1484	cysteine dioxygenase type I
colony3	1993126	A	G	131	0	0.000	Francci3_1658	hypothetical protein
colony3	2195005	G	T	78	3	0.038	Francci3_1861	2-dehydropantoate 2-reductase
colony3	2318177	T	C	54	3	0.056	Francci3_1976	amino acid adenylation
colony3	2553579	T	C	1549	37	0.024	Francci3_2179	hypothetical protein
colony3	2801764	T	C	179	5	0.028	Francci3_2413	major facilitator transporter
colony3	2918202	T	C	1051	33	0.031	IGR|Francci3_2473-Francci3_2474	
colony3	3425853	C	G	4	1	0.250	Francci3_2909	peptidase S9, prolyl oligopeptidase active site region
colony3	3999160	A	G	69	2	0.029	Francci3_3370	cobalamin B12-binding
colony3	4318977	T	C	539	13	0.024	Francci3_3617	diguanylate cyclase/phosphodiesterase
colony3	4425344	T	C	76	2	0.026	Francci3_3688	hypothetical protein
colony3	4631958	T	C	97	3	0.031	Francci3_3869	hypothetical protein
colony3	5322012	A	G	134	2	0.015	Francci3_4451	acetyl-CoA acetyltransferase
colony4	140311	T	C	102	1	0.010	Francci3_0114	phage integrase
colony4	424544	G	C	5	1	0.200	Francci3_0362	hypothetical protein
colony4	755715	T	C	86	6	0.070	Francci3_0660	geranylgeranyl reductase
colony4	887559	T	C	7	1	0.143	Francci3_0762	putative integral membrane protein
colony4	1116519	T	C	570	11	0.019	Francci3_0947	FHA domain-containing protein
colony4	1177025	A	G	573	21	0.037	Francci3_0991	acyl transferase region
colony4	1446690	T	C	447	19	0.043	Francci3_1211	rod shape-determining protein MreB
colony4	1752341	C	G	4	1	0.250	Francci3_1460	peptidase C60, sortase A and B
colony4	1776439	A	G	62	4	0.065	Francci3_1484	cysteine dioxygenase type I
colony4	1790485	T	C	182	4	0.022	IGR|Francci3_1495-Francci3_1496	

Strain	Position (nt)[a]	Mutant base	WT base[b]	Coverage[c]	No. WT base[d]	Ratio of WT base[e]	ID[f]	Annotation
colony4	2078892	A	G	1025	21	0.020	Francci3_1745	hypothetical protein
colony4	2206561	T	C	230	7	0.030	Francci3_1871	hypothetical protein
colony4	2247733	C	T	23	2	0.087	Francci3_1913	OmpA/MotB
colony4	2801764	T	C	127	5	0.039	Francci3_2413	major facilitator transporter
colony4	3148274	A	G	139	1	0.007	Francci3_2666	MMPL
colony4	3473687	G	C	5	1	0.200	Francci3_2940	protein of unknown function DUF1524 RloF
colony4	4193176	A	G	242	7	0.029	Francci3_3508	transcriptional regulator NrdR
colony4	4318977	T	C	358	10	0.028	Francci3_3617	diguanylate cyclase/phosphodiesterase
colony4	4634847	C	A	4	1	0.250	Francci3_3872	hypothetical protein
colony4	4677327	G	C	7	0	0.000	Francci3_3908	hypothetical protein
colony5	54308	A	G	103	10	0.097	Francci3_0044	serine/threonine protein kinase
colony5	141500	A	G	1675	67	0.040	Francci3_0114	phage integrase
colony5	498466	A	G	812	6	0.007	IGR|Francci3_0419-Francci3_R0012	
colony5	629808	T	C	193	6	0.031	Francci3_0543	NADH-quinone oxidoreductase, F subunit
colony5	1450426	T	C	15	2	0.133	Francci3_1214	peptidoglycan glycosyltransferase
colony5	1515535	T	C	31	1	0.032	Francci3_1269	CBS
colony5	1943172	T	C	123	3	0.024	Francci3_1619	extracellular ligand-binding receptor
colony5	1960376	T	C	1096	43	0.039	Francci3_1631	excinuclease ABC subunit A
colony5	1965419	C	T	6	1	0.167	Francci3_1633	excinuclease ABC subunit C
colony5	2106877	A	G	147	9	0.061	IGR|Francci3_1770-Francci3_1771	
colony5	2195005	G	T	28	4	0.143	Francci3_1861	2-dehydropantoate 2-reductase
colony5	2316337	A	G	985	46	0.047	IGR|Francci3_1975-Francci3_1976	
colony5	2458991	A	G	110	8	0.073	Francci3_2090	3-oxoacyl-(acyl carrier protein) synthase III
colony5	2930887	A	G	1865	43	0.023	IGR|Francci3_2484-Francci3_2485	
colony5	2959308	T	C	1266	24	0.019	Francci3_2506	hydantoinase B/oxoprolinase
colony5	3211711	A	G	157	6	0.038	Francci3_2727	hypothetical protein
colony5	3717464	A	G	242	4	0.017	Francci3_3143	L-glutamine synthetase
colony5	5202265	T	C	6	1	0.167	Francci3_4356	ATPase AAA-2
colony6	1712679	A	G	90	1	0.011	Francci3_1421	hypothetical protein
colony6	2440561	A	G	147	3	0.020	Francci3_2075	aminotransferase
colony6	3362356	T	C	221	4	0.018	Francci3_2851	beta-ketoacyl synthase
colony6	3444812	T	C	149	7	0.047	IGR|Francci3_2923-Francci3_2924	

Strain	Position (nt)[a]	Mutant base	WT base[b]	Coverage[c]	No. WT base[d]	Ratio of WT base[e]	ID[f]	Annotation
colony6	3610715	T	C	295	13	0.044	Francci3_3042	acyl-CoA dehydrogenase-like
colony6	3729412	A	G	35	1	0.029	Francci3_3150	hypothetical protein
colony6	3937737	C	G	3	1	0.333	Francci3_3323	hypothetical protein
colony6	4384188	T	C	369	9	0.024	IGR|Francci3_3662-Francci3_3663	
colony6	4511418	G	A	5	1	0.200	Francci3_3762	dithiobiotin synthetase
colony6	4630236	T	C	49	0	0.000	Francci3_3866	NUDIX hydrolase
colony6	4649421	T	C	315	15	0.048	IGR|Francci3_3885-Francci3_3886	
colony6	4814994	T	C	666	7	0.011	Francci3_4027	LuxR family transcriptional regulator
colony7	230604	A	G	114	0	0.000	Francci3_0194	hypothetical protein
colony7	491327	T	C	64	1	0.016	Francci3_0412	cell divisionFtsK/SpoIIIE
colony7	755715	T	C	78	0	0.000	Francci3_0660	geranylgeranyl reductase
colony7	948422	A	G	167	0	0.000	Francci3_0815	NUDIX hydrolase
colony7	1116519	T	C	87	2	0.023	Francci3_0947	FHA domain-containing protein
colony7	1431150	A	G	3	1	0.333	Francci3_1199	putative ATP-binding protein
colony7	1703462	A	G	31	0	0.000	Francci3_1413	UDP-N-acetylmuramoyl-L-alanyl-D-glutamate synthetase
colony7	1776439	A	G	43	1	0.023	Francci3_1484	cysteine dioxygenase type I
colony7	1853798	A	G	231	2	0.009	IGR|Francci3_1543-Francci3_1544	
colony7	2801764	T	C	53	0	0.000	Francci3_2413	major facilitator transporter
colony7	3621843	A	G	63	0	0.000	IGR|Francci3_3055-Francci3_3056	
colony7	3714154	A	G	8	0	0.000	IGR|Francci3_3140-Francci3_3141	
colony7	3816051	T	C	83	1	0.012	Francci3_3221	hypothetical protein
colony7	4318977	T	C	155	2	0.013	Francci3_3617	diguanylate cyclase/phosphodiesterase
colony8	498466	A	G	167	0	0.000	IGR|Francci3_0419-Francci3_R0012	
colony8	550578	G	C	10	1	0.100	Francci3_0470	histidine kinase
colony8	629808	T	C	43	0	0.000	Francci3_0543	NADH-quinone oxidoreductase, F subunit
colony8	1132805	A	G	121	2	0.017	Francci3_0959	hypothetical protein
colony8	1301931	A	T	12	0	0.000	Francci3_1089	transposase, IS4
colony8	2316337	A	G	168	1	0.006	IGR|Francci3_1975-Francci3_1976	
colony8	2424161	T	C	56	2	0.036	IGR|Francci3_2059-Francci3_2060	
colony8	2424201	T	C	9	1	0.111	Francci3_2060	hypothetical protein
colony8	2930887	A	G	397	2	0.005	IGR|Francci3_2484-Francci3_2485	
colony8	3211711	A	G	59	1	0.017	Francci3_2727	hypothetical protein

Strain	Position (nt)[a]	Mutant base	WT base[b]	Coverage[c]	No. WT base[d]	Ratio of WT base[e]	ID[f]	Annotation
colony8	4381115	T	C	8	1	0.125	Francci3_3660	putative transcriptional regulator
colony8	4457541	C	G	6	1	0.167	Francci3_3723	homoserine kinase
colony8	4903846	A	G	71	6	0.085	IGR|Francci3_4094-Francci3_4095	
colony8	5391784	G	C	6	1	0.167	Francci3_4516	hypothetical protein
colony9	498466	A	G	516	1	0.002	IGR|Francci3_0419-Francci3_R0012	
colony9	629808	T	C	200	0	0.000	Francci3_0543	NADH-quinone oxidoreductase, F subunit
colony9	880957	A	G	417	2	0.005	Francci3_0757	extracellular solute-binding protein
colony9	1564048	T	C	259	0	0.000	Francci3_1306	oxidoreductase-like
colony9	1582887	A	G	119	0	0.000	Francci3_1319	hypothetical protein
colony9	2316337	A	G	662	5	0.008	IGR|Francci3_1975-Francci3_1976	
colony9	2930887	A	G	1121	12	0.011	IGR|Francci3_2484-Francci3_2485	
colony9	3042202	A	G	124	3	0.024	Francci3_2576	hypothetical protein
colony9	3211711	A	G	140	0	0.000	Francci3_2727	hypothetical protein
colony9	3425584	C	G	5	0	0.000	Francci3_2909	peptidase S9, prolyl oligopeptidase active site region
colony9	4823021	C	A	6	0	0.000	Francci3_4037	molybdopterin adenylyltransferase
colony9	5129264	C	G	12	0	0.000	Francci3_4298	type II secretion system protein
shiro1	498466	A	G	427	0	0.000	IGR|Francci3_0419-Francci3_R0012	
shiro1	1208408	T	C	217	9	0.041	Francci3_1014	hypothetical protein
shiro1	2316337	A	G	511	6	0.012	IGR|Francci3_1975-Francci3_1976	
shiro1	2930887	A	G	851	10	0.012	IGR|Francci3_2484-Francci3_2485	
shiro1	3026410	A	G	256	4	0.016	Francci3_2567	putative hydrolase
shiro1	3211711	A	G	98	3	0.031	Francci3_2727	hypothetical protein
shiro1	3235654	A	G	35	0	0.000	Francci3_2745	hypothetical protein
shiro2	498466	A	G	564	1	0.002	IGR|Francci3_0419-Francci3_R0012	
shiro2	629808	T	C	121	2	0.017	Francci3_0543	NADH-quinone oxidoreductase, F subunit
shiro2	1423887	G	C	3	1	0.333	Francci3_1190	hypothetical protein
shiro2	2285845	C	G	11	0	0.000	Francci3_1945	(NiFe) hydrogenase maturation protein HypF
shiro2	2316337	A	G	628	7	0.011	IGR|Francci3_1975-Francci3_1976	
shiro2	2768445	A	G	135	1	0.007	Francci3_2390	glycosyl transferase family protein
shiro2	2930887	A	G	1213	10	0.008	IGR|Francci3_2484-Francci3_2485	
shiro2	3211711	A	G	114	0	0.000	Francci3_2727	hypothetical protein
shiro2	3235654	A	G	45	2	0.044	Francci3_2745	hypothetical protein

Strain	Position (nt)[a]	Mutant base	WT base[b]	Coverage[c]	No. WT base[d]	Ratio of WT base[e]	ID[f]	Annotation
shiro2	3714154	A	G	9	0	0.000	IGR|Francci3_3140-Francci3_3141	
shiro2	4949435	G	T	5	0	0.000	Francci3_4149	integrase
shiro3	398331	A	G	586	18	0.031	Francci3_0343	inner-membrane translocator
shiro3	755715	T	C	127	2	0.016	Francci3_0660	geranylgeranyl reductase
shiro3	1049059	A	C	135	2	0.015	Francci3_0906	Type IV secretory pathway VirD4 components-like
shiro3	1116519	T	C	398	4	0.010	Francci3_0947	FHA domain-containing protein
shiro3	1183365	A	G	1059	29	0.027	Francci3_0994	hypothetical protein
shiro3	1408614	T	C	137	6	0.044	Francci3_1178	AMP-dependent synthetase and ligase
shiro3	1868217	G	T	6	1	0.167	Francci3_1558	cell envelope-related transcriptional attenuator
shiro3	2145810	A	G	232	5	0.022	Francci3_1810	uncharacterized FAD-dependent dehydrogenase
shiro3	2161726	T	C	139	2	0.014	Francci3_1823	putative O-methyltransferase
shiro3	2801764	T	C	146	1	0.007	Francci3_2413	major facilitator transporter
shiro3	3174296	A	G	360	9	0.025	Francci3_2688	Rieske (2Fe-2S) protein
shiro3	3267212	T	C	124	1	0.008	Francci3_2770	4-hydroxyphenylacetate 3-hydroxylase
shiro3	4309260	T	C	269	5	0.019	Francci3_3607	DEAD/DEAH box helicase-like
shiro3	4318977	T	C	355	2	0.006	Francci3_3617	diguanylate cyclase/phosphodiesterase
shiro3	4672005	A	G	641	9	0.014	IGR|Francci3_3903-Francci3_3904	

[a]Position of the base in the reference genome sequence of *Frankia* CcI3 in public database (NC_007777) .

[b]Base reported in the reference genome sequence.

[c]Tolal number of reads mapped on the base.

[d]The number of reads that showed WT base.

[e]No. WT base / Coverage.

[f]IGR represents ingergenic region.
